# Gender role identity, personality factors, and psychiatric symptoms among American adults: the Nathan Kline Institute Rockland Sample

**DOI:** 10.3389/fpsyt.2025.1594762

**Published:** 2025-09-26

**Authors:** Mathias Rossi, Maryse Arcand, Mike Schmidt, Theodorus G. M. Sandfort, Francelyne Jean-Baptiste, Marie-France Marin, Spiro P. Pantazatos, Robert-Paul Juster

**Affiliations:** ^1^ Research Center, Institut Universitaire en Santé Mentale de Montréal, Montreal, QC, Canada; ^2^ Department of Psychiatry and Addiction, University of Montreal, Montreal, QC, Canada; ^3^ Molecular Imaging and Neuropathology Division, New York State Psychiatric Institute, New York, NY, United States; ^4^ Department of Psychiatry, Columbia University Medical Center, New York, NY, United States; ^5^ HIV Center for Clinical and Behavioral Studies, New York State Psychiatric Institute and Columbia University, New York, NY, United States; ^6^ Interdisciplinary Research Centre on Intimate Relationship Problems and Sexual Abuse, Montreal, QC, Canada; ^7^ Department of Psychology, Université du Québec à Montréal, Montreal, QC, Canada

**Keywords:** gender roles, personality traits, anxiety, depression, suicide

## Abstract

**Introduction:**

Gender roles and personality traits have been reported to impact mental health. This study aims to investigate the relationship between gender role identity and psychiatric symptoms (anxiety, depressive symptoms, suicidality) as well as the moderating effects of personality traits in a community-representative sample of American adults.

**Methods:**

Data from 741 participants (65.7% females) were analyzed from the Nathan-Kline Institute – Rockland Sample database, a community-ascertained lifespan cohort with participants undergoing multimodal brain imaging and comprehensive behavioral, cognitive, and psychiatric assessments. This analysis is restricted to adults and uses well-validated questionnaires to assess gender role identity, personality traits, symptoms of anxiety and depression, and suicidal thoughts/behaviors.

**Results:**

Results revealed that having a gender role identity reversed to one’s birth-assigned sex (i.e., feminine gender role in males and masculine gender role in females) was associated with poorer mental health (i.e., more anxiety and depressive symptoms). This effect was stronger in males where femininity was positively associated with more suicidal thoughts and behaviors. Further analyses revealed that only low-extroverted feminine males reported higher anxiety, and only high-neurotic feminine males reported higher suicidality.

**Conclusions:**

The present American study provides new understanding on gender role identity associations with mental health, while highlighting the importance of considering both birth-assigned sex and personality traits when studying gender role effects on psychiatric symptoms. We discuss the role of gendered traits and societal burden in relation to mental health.

## 1 Introduction

Sex differences are a major consideration for mental health disorders (1). For instance, women engage more frequently in suicidal behaviors, whereas men are more likely to complete suicide (2). This “gender paradox” is consistent in the literature (3–5), and is also common in other psychiatric conditions like depression and anxiety: girls report an increasing rate of depressive symptoms and anxiety higher than boys from adolescence onwards (6, 7). Studies have also reported that symptoms of anxiety and depression, which are highly comorbid, can contribute strongly to suicidal symptoms (8–10). Women show higher rates of anxiety and depression than men in adulthood (11, 12). This can contribute to their higher rate of suicide attempts. By contrast, men are less likely to report or seek help for mental health issues while using more lethal approaches, which ultimately contributes to higher rates of completed suicide (13). While biological factors have also been investigated to explain sex differences in suicide rates (14) and the two-times higher prevalence of depression in females (15), environmental factors (11) like socio-economic status (16), major life events (5), or personality traits (17) also play a key role in the prevalence of anxious, depressive, and suicidal symptoms. Beyond sex as a biological binary, the current research aims to understand the role of socio-cultural gender role identity in association with psychiatric symptoms and suicidality (i.e. suicidal thoughts and behaviors) (18).

### 1.1 Socio-cultural gender roles

Several studies have reported that gender roles explain variance in anxiety and depression beyond that of biological sex (19–21). Gender is a multidimensional concept that includes sociocultural roles and identities (i.e., men, women, nonbinary people) that are different from one’s biological sex (i.e. sex assigned at birth: male, female, intersex) that is otherwise determined by genes, hormone levels, and gonads (22, 23). While growing up and socializing, children will endorse sex-specific personality traits or gender roles by performing behaviors that are stereotypically classified as “feminine” (e.g. affective concerns) and “masculine” (e.g. cognitive focus on action) (24). Several questionnaires, like the Personal Attributes Questionnaire (PAQ) (25), have aimed to assess one’s femininity and masculinity by analyzing personality traits and classifying their descriptions as “feminine” or communal and “masculine” or agentic factors.

In the 1970s, Bem developed the Bem Sex Role Inventory (BSRI) that assesses femininity and masculinity on two orthogonal but still complementary continuums (26). Two approaches to gender roles have been developed. First, the categorical method that classifies people as masculine, feminine, androgynous (when both of their feminine and masculine scores are high), or undifferentiated (when both of their scores are low). Second, a continuous method that considers one’s sex and mixture of feminine and masculine continuums to obtain diverse gender role profiles. For instance, people can be sex-typed (when their gender role profile matches their birth-assigned sex), cross-typed (when their gender role profile is opposed to their birth-assigned sex), or mix-typed (when their gender role profile is not mainly feminine nor masculine). As gender roles develop across the lifespan and in the face of stressful life events, they may modulate the way mental health problems manifest themselves (27). Indeed, gender roles overlap with personality traits that can synergistically influence coping strategies (e.g. emotional regulation, self-esteem, adjustment) that are collectively associated with anxiety, depression, and even suicidal behaviors (28).

### 1.2 Gender roles, anxiety and depression

Whether it has been measured with the PAQ or the BSRI, higher masculinity has been consistently associated with fewer reported symptoms of depression and anxiety (20, 21, 29–32). However, femininity has been less consistently associated with mental health. Some studies have shown that higher femininity is related to more general distress (33), anxiety and depressive symptoms (21, 34). On the other hand, several studies have not shown a significant effect of femininity on mental health (30–32, 35–37), and some even found mixed results where femininity was related to fewer depressive symptoms (29, 38). A meta-analysis by Whitley in 1985 concluded that higher masculinity was associated with less depression and greater adjustment, whereas femininity had no consistent relationship with depression (37).

The apparent heterogeneity of results linking feminine gender roles to depression may be due to different ways that gender roles operate among men and women (20). Some studies did not find significant differences between the way that gender roles affect sex differences in mental health (36). By contrast, several studies have highlighted mixed associations between gender roles and mental health. For example, a study by Gibson et al. reported that higher femininity was associated with fewer depressive symptoms in college educated men, while masculinity had no effect only for women. This highlights the need to consider other demographic and socio-cultural factors such as education level in the study of gender roles (38).

Some studies have investigated the effect of gender roles only in one sex or specific race/ethnic groups. Among females, higher femininity has been associated with more anxiety and depression (39, 40), while less masculinity has been related to more depressive symptoms (30). However, for Black women, higher femininity has been associated with less depressive symptoms (41), which underlines again the inconsistency of findings among females, as well as the importance of considering race/ethnicity and intersecting sociodemographics more broadly in gender role research (42).

### 1.3 Gender roles and suicidality

It has been proposed that higher masculinity decreases self-harm only in males (43). However, some sociocultural profiles related to certain masculine norms such as self-reliance, difficulty expressing emotions and reduced help-seeking behavior may be ‘maladaptive’ with respect to men’s mental health, particularly in developed Western societies where men experience decreasing social and economic role opportunities (real or perceived) since the 1960s (44, 45). Indeed, higher male-to-female suicide ratios in more common in highly developed, gender egalitarian nations (46). In addition, a study of men hospitalized following stressful life events found that adherence to traditional male gender roles mediated previous suicide attempt status in men (47). Beyond extreme masculinity and femininity alone, it appears that the extent that one’s gender role concords with one’s birth-assigned sex is also a unique predictor of suicide risk. Indeed, cross-typed people (i.e., people with a gender role or personality typology opposed to their birth-assigned sex) are considered to be at higher risk of suicidal symptoms than sex-typed people (i.e., people with a gender role or personality typology in accordance with their birth-assigned sex) (48). These results emphasize that even if gender roles are better predictors of psychological health than other factors like sex (19–21, 49) or sexual orientation (48, 50), it is still important to take birth-assigned sex into account to better understand how gender roles influence well-being as gender roles and personality operate differently for males and females (20).

### 1.4 Considerations regarding gender role assessments

The apparent heterogeneity in the literature could be due to several methodological issues including the sample sizes and the way gender roles are conceptualized, operationalized and assessed. Indeed, even if gender roles seem to be consistent across generations, Adams and Sherer suggested in 1985 that classic inventories of gender role like PAQ or BSRI measure constructs like assertiveness and self-efficacy better than gender roles (51). Besides, even if Bem had the original intention to measure gender roles without a negative desirability bias, a study by Grimmell and Sterne (1992) reported that the Bem feminine scale of the BSRI contains both positively and negatively valued traits (52). That is, classic measures of gender roles can suffer from a social-desirability bias that affects the way participants evaluate their stereotyped personality traits. This valuation of their own traits may confound mental health measurement. For example, one group showed that only negatively evaluated aspects of femininity of the Australian Sex Role Scale were associated with suicidality (53). More recent studies indicate that most people nowadays disagree with the “masculine” and “feminine” classifications of some characteristics described by the BSRI, suggesting the need of an alternative way to evaluate gender roles which does not suffer from a time period bias (54).

### 1.5 Self-identified gender roles or gender role identity

In 1979, Storm created a shortened questionnaire that directly asks participants how they feel about themselves regarding their masculinity and femininity (55) with a simple measure of gender role identity. In this manner, participants fall somewhere on two dimensions comprising their self-perception and perception of others’ perception regarding their masculinity and femininity. Participants are asked to evaluate how they feel, act, and appear to others as more or less feminine and masculine without evoking concepts that can bias their answer regarding both gender role and mental health.

Several studies have underlined the importance of other factors that influence the relationship between gender roles and mental health beyond the questionnaire used. For instance, Sandfort et al. (2021) showed in a US national sample that gender conformity is more present among people with less social status (e.g., age, race/ethnicity, education, income) (56). Gender non-conformity can in turn negatively affect mental health. Taken together, the effects of gender roles on mental health differ according to student/worker status (29), education level (38), race/ethnicity (57), or self-esteem (21, 31). Age also seems to play a key role that influences the effect of gender roles on mental health. For instance, Hunt et al. only succeeded in detecting a significant effect of gender roles on suicidal thoughts among early middle-age participants, but not in early adulthood or late middle-age (58).

### 1.6 Personality traits and mental health

Beyond age, sex and race/ethnicity, personality traits are another facet of psychosocial functioning that, like gender roles, can influence mental health and behaviors (59–61). The classic NEO-Five factor inventory (62) breaks down personality into five traits: openness, conscientiousness, extroversion, agreeableness, and neuroticism. The link between these personality traits and mental health has been investigated by numerous studies. Overall, it appears that neuroticism, which represents our vulnerability to experience negative affects including anger and emotional instability, is the only trait that strongly and consistently predicts higher anxiety and depressive symptoms for both sexes (63–68). In contract to neuroticism, extroversion, which is the trait that depict enjoyment for activities involving social interactions, seems to correlate with lower anxiety and depression (63, 65).

In addition, different factors are associated with gender roles, like perceived stress (28) or rumination (69), mediate the relationship between neuroticism and anxiety and depressive symptoms (66, 67). Wupperman and Neumann found that masculinity was associated with lower neuroticism (69), highlighting the close relationship between gender roles, mental health and personality traits. It is no surprise then that personality traits also affect suicidality. Indeed, neuroticism, just like for anxiety and depression, strongly and consistently predicts higher suicidal thoughts (17, 70–74) and behaviors (75) regardless of sex (71).

The link between the other traits and suicidality is less clear. The most consistent evidence suggests that lower levels of extroversion (17, 71, 72, 74) and conscientiousness (70, 71, 74) predicts higher rates of suicidal thoughts. A recent review by Szücs highlighted the importance of age in this relationship between personality traits and suicidality. Especially, elderly people who committed suicide displayed lower levels of openness than younger victims (76). As a whole, personality traits can influence the relationship between gender roles and mental health, as they do for sex and depression (77), since they greatly influence how people feel and act, as well as their psychological health.

### 1.7 Objectives and hypotheses of the present study

To date, gender roles have been associated with mental health outcomes, but results are not consistent, especially regarding the manner in which masculinity and femininity are contextualized and operationalized. Indeed, these mixed results may be due to the methods used. Specifically, some studies have not disaggregated analysis by sex as recommended for rigor and reproducibility in health research (78), and gender roles have mostly been measured by questionnaires that used concepts that may bias participants’ answers. In addition, personality traits should be considered in research on gender roles since they may synergize associations with mental health outcomes.

The present study aims to investigate how self-identified gender roles relate to anxiety, depressive and suicidal symptoms in a well-phenotyped sample drawn from the general population. In sex-specific analyses, we also assess whether personality traits moderate the aforementioned associations. *A priori* covariates like age, race and ethnicity are also accounted for.

In analyses split by sex, we hypothesize that self-reported femininity will be associated with poorer mental health, as evidenced by higher reported anxiety, depression, and suicidal symptoms. By contrast, masculinity will be associated with lower reported anxiety, depression and suicidal thoughts and behavior. Also, in agreement with the literature, we hypothesize that gender role identity’s effect on mental health will be different in males and females. Specifically, we hypothesize that “cross-typed” profiles (i.e., feminine males and masculine females) will present poorer mental health, while “sex-typed” people will report fewer symptoms of anxiety, depression, and suicide. However, since there is insufficient literature on the role of personality traits as a moderator of associations between gender roles and mental health, we have not explicitly hypothesized directionality for this exploratory hypothesis.

## 2 Methods

### 2.1 Participants

This analysis used data from the Nathan Kline Institute Rockland Sample (NKI-RS) (79). The larger NKI-RS project is a comprehensive community sample of participants studied across the lifespan. Efforts have been concentrated on recruitment strategies to avoid over-representation of any portion of the Rockland County community. Recruitment flyers were posted at schools, shopping malls, community centers, and various other locations in Rockland County in New York State. The resulting sample of more than 1000 participants was recruited between 2012 and 2016. As race, ethnicity and economic demographics of Rockland County are similar to those of the United States, the NKI-RS is generalizable to the broader U.S. population. The larger study also includes the collection of rich phenotyping as well as advanced neuroimaging data. For this study, we restricted our analyses to self-reported behavioral data among adults ages 18 and above.

Participants under 18 years old were excluded (n = 155) as the Trait Anxiety Standard Score was only validated and collected for people above 18 years old. Participants with missing data were also excluded (n = 427). In the current analysis, 741 participants (487 females and 254 males) were therefore included from the NKI-RS.

### 2.2 Ethics statement

Institutional Review Board (IRB) approval was obtained for the original project at the Nathan Kline Institute (Phase I #226781 and Phase II #239708) and at Montclair State University (Phase I #000983A and Phase II #000983B). Written informed consent was obtained for all study participants. An additional IRB approval for our analyses of these data was not required. Instead, we reviewed and signed the NKI-RS Data Usage Agreement.

### 2.3 General protocol

Data used in this analysis are part of larger dataset mentioned above. For further information, please refer to the NKI-Rockland sample description by K. B. Nooner et al. (79). All participants took part in a 2-day experiment composed of biological data measurements, interviews, questionnaires, magnetic resonance imaging, various cognitive tasks, and behavioral measures. Demographic and gender role identity data were collected at the arrival on the first day (8:15 AM), while all the other data used in this analysis were collected during the one-hour questionnaire phase starting at 1:45 PM the same first day.

### 2.4 Measures

#### 2.4.1 Demographics

Birth-assigned sex (male/female), age, race, and ethnicity were self-reported. Participants had a mean age of 47.82 years old (SD = 0.66) and were mainly females (65.7%). The sample comprised of White (77.1%), Black (14.3%), Asian (4.9%), American Indian (0.9%), and other-race participants (2.8%). To conduct our analyses, race was turned into a dichotomous variable: White (77.1%) and Non-White (22.9%). Ethnicity is also coded as a dichotomous variable: Hispanic (10.5%) and Non-Hispanic (89.5%).

#### 2.4.2 Gender role identity

A shortened version of the Sex [Gender] Role Identity Scale (GRIS) was used to assess participants’ gender role identity. This 30 item-scale was developed by M. D. Storms in 1979 and has an internal consistency ranging from 0.66 to 0.80 (55). A shortened version of this scale was used to assess how people perceive themselves or think that they are perceived as more feminine and/or masculine. This scale is composed of 6 items, 3 about masculinity and 3 about femininity: “In general, how [masculine] feminine do you think you are?”, “In general, how [masculine] feminine do you think you act or behave?”, and “How [masculine] feminine do you think you appear or come across to others?”. This scale showed strong internal consistency, with the three masculine identity items inter-correlating positively for men (r = .66, p <.001) and for women (r = .68, p <.001) and the three feminine identity items inter-correlating positively for men (r = .80, p <.001) and for women (r = .70, p <.001; Storms, 1979). In the current sample, internal consistency is high for the masculinity sub-scale (Cronbach’s α = .958) and the femininity sub-scale (Cronbach’s α = .969).

**Figure 1:**
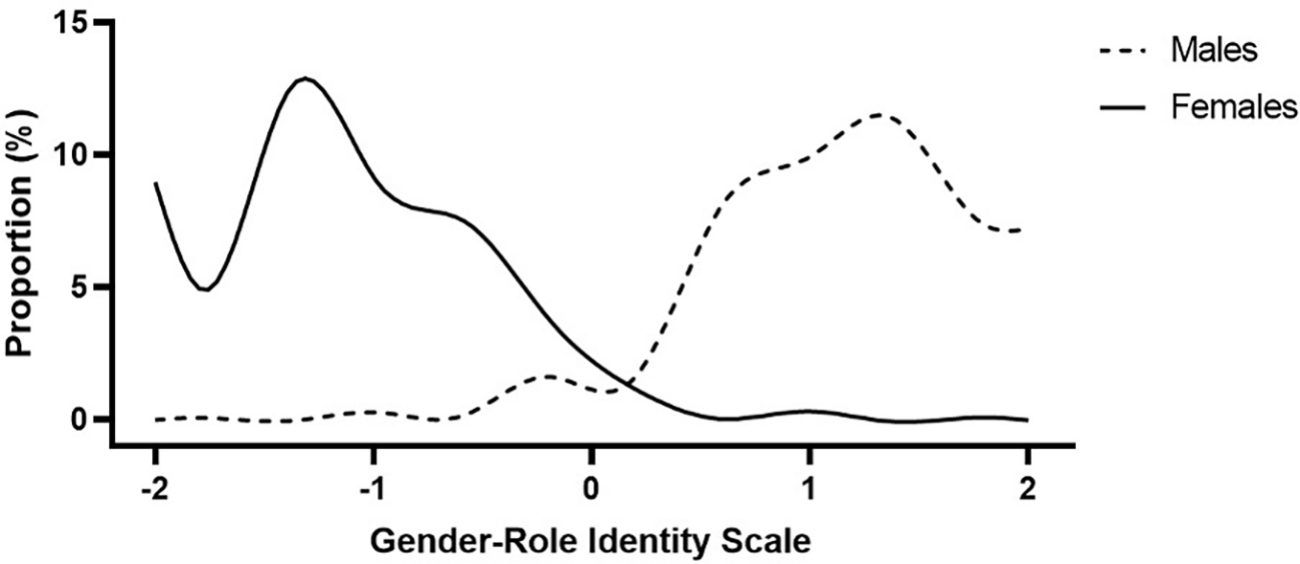
Smoothing splines (12 knots) of the gender role identity scale (GRIS) index distribution among males and females (N = 741). More negative scores represent greater femininity relative to masculinity, and more positive scores represent greater masculinity relative to femininity.

Every item is coded on a 5 point-scale: 1 “Not at all”, 2 “Very little”, 3 “Fairly”, 4 “Very much”, 5 “Extremely”, and two scores are then calculated to obtain a femininity score (mean of the 3 feminine items) and a masculinity score (mean of the 3 masculine items) separately. For example, a high score on masculinity and a low score on femininity means that the participant considers that they are and appear to others as high masculine but low feminine. Next, these 2 scores are grouped to form the GRIS index according to the following formula $GRIS=\frac{\text{Masculinity score }-\text{ Femininity score}}{2}$ , ranging from -2 (very feminine) to +2 (very masculine). In this manner, a more negative score represents greater femininity relative to masculinity while a more positive score represents greater masculinity relative to femininity. This combined approach allows us to eliminate any issues of multi-collinearity by combining the Masculinity score and the Femininity score as a single GRIS index (**Figure 1**).

#### 2.4.3 Personality factors

The NEO-FFI is a 60 item self-rating personality assessment instrument developed to provide a more concise measurement of the five personality factors captured in the Revised NEO Personality Inventory (80). The five factors assessed by this instrument include Openness to Experience, Conscientiousness, Extroversion, Agreeableness, and Neuroticism. Participants are asked to choose the answer that represents their opinion on a 5-point Likert scale: 0-Strongly Agree, 1-Agree, 2-Neutral, 3-Disagree, 4-Strongly Disagree for each of the 60 items. Five scores are obtained, one for every personality factor. The original questionnaire has an internal consistency that ranges from 0.68 to 0.86. The test-retest reliability coefficients ranged from .86 to .90 (80).

#### 2.4.4 Mental health

##### 2.4.4.1 Trait anxiety standard score

Participants completed the 40-item State Trait Anxiety Inventory that assesses anxiety in adults ages 18 to 85 (81). Divided into two sections of 20 questions, it measures state and trait anxiety. Both sections use a 4-point scale rating: 1 Almost never, 2 Sometimes, 3 Often, 4 Almost always. A higher score corresponds with greater symptoms of anxiety. The questionnaire has an internal consistency ranging from.86 to.95. The test-retest reliability also ranges from .65 to .75 over an interval of 2 months (82).

##### 2.4.4.2 Beck depression inventory

Participants completed the 21-item Beck Depression Inventory II. This questionnaire aimed at assessing the severity of typical symptoms of depression such as mood, pessimism, self-dislike, and insomnia. Participants had to choose an answer on a 4-point scale that best describes how they have been feeling during the past two weeks. A higher score corresponds to greater symptoms of depression. The questionnaire has a strong internal consistency coefficient of .91 (83), as well as an high test-retest reliability coefficient of .93 (84).

##### 2.4.4.3 Suicidality

Suicidality was assessed by the combination of two scores assessing suicidal ideation and suicide attempts. Suicidal ideation was assessed using self-report measures from the Adult Self-Report (ages 18-59) and the Older Adult Self-Report (ages 60+). Each participant was administered the age-appropriate assessment, which included the statement, “I think about killing myself.” Participants endorsed either “not true”, “somewhat or sometimes true” or “very true or often true.” Suicidal ideation was then binary coded as “yes” if the participant answered “somewhat or sometimes true” or “very true or often true”, or “no” if the participant answered “not true”. Suicide attempt/self-harm was assessed using self-report measures (i.e., the Adult Self-Report and the Older Adult Self-Report) based on responses to the statement “I deliberately try to harm or kill myself.” Participants endorsed either “not true”, “somewhat or sometimes true”, or “very true or often true.” Suicidal behaviors were then binary coded as “yes” or “no” in the same way as for suicidal thoughts. Additionally, participants who indicated a history of suicide attempt in their medical history were included.

Suicide scores were then coded as an ordinal variable. Participants with no suicidal ideation and behavior were coded as “0”, “1” for suicidal thoughts only, “2” for suicidal thoughts and behaviors. Considering the low rate of people with “1” (n = 31) and “2” (n = 10) scores, suicide score will be analyzed as a continuous variable in the following analyses.

### 2.5 Statistical analyses

Statistical analyses were conducted using IBM Statistical Package for the Social Sciences (SPSS) (version 26). Considering the large sample size of the NKI-RS and that sex is an important factor for every variable, all analyses were split by sex as recommended by Clayton and Tannenbaum for improving rigor and reproducibility (78). The sample sizes and degrees of freedom for each statistical model differ slightly since not all measures were collected from all participants at the same time.

#### 2.5.1 Preliminary analyses

First, we conducted independent sample t-tests for personality traits, gender role identity, anxiety symptoms, depressive symptoms, and suicide scores to investigate if there are sex differences in these key study variables. Then, we conducted correlation analyses between our 14 variables in females and males separately for descriptive purposes. To investigate the effect of gender role identity scores (GRIS index, Masculinity score, Femininity score) on personality traits, we also conducted three separate multivariate analysis of variance (MANOVA) in females and males separately (see results in the **Supplementary**). Finally, we conducted multiple linear regression analysis with TAS, BDI, and suicide scores as dependent variables to investigate the effect of personality traits on mental health in females and males separately. We adjusted for age, race, and ethnicity for this last preliminary analysis that is reported next.

#### 2.5.2 Main analyses

First, to assess the effect of gender role identity scores on mental health, we conducted multiple linear regression analyses with GRIS index, Femininity scores, and Masculinity scores as independent variables in females and males separately. Secondly, to investigate if there is an interaction effect between personality traits and gender roles on mental health, we performed moderation analyses using PROCESS macro for SPSS (85) with gender roles as predictor and each personality trait, which appeared significant in the precedent analyses, as observed in females and males separately. When two personality traits appeared to be significant predictors of mental health variables in multiple linear regression analyses, we used one of them as a moderator and the other one as a covariable, then vice versa, in moderation analyses. We adjusted for age, race, and ethnicity for every main analysis.

## 3 Results

### 3.1 Preliminary analyses

#### 3.1.1 Sex differences

**Tables 1** and **2** present correlations between age, gender role identity, personality traits, and mental health for males and females. T-tests showed no significant sex differences in conscientiousness (t(460) = -0.050, p = .960), agreeableness (t(734) = 0.367, p = .714), depression (t(624) = -0.682, p = .495), or suicide scores (t(461) = 1.361, p = .174). Significant differences emerged for openness (t(732) = 4.62, p <.001), extroversion (t(453) = 2.41, p = .016), neuroticism (t(737) = 2.464, p = .014), GRIS (t(739) = 48.78, p <.001), masculinity (t(739) = 40.40, p <.001), femininity (t(603) = -45.95, p <.001), and anxiety (t(576) = -2.38, p = .018).

**Table 1:** Correlation matrix for males.

Variable	*M (SE)*	Correlation coefficients												
		1.	2.	3.	4.	5.	6.	7.	8.	9.	10.	11.	12.	13.
1. Age	46.25 (1.21)	–												
2. Race	–	0.357***	–											
3. Ethnicity	–	-0.145*	-0.043	–										
4. GRIS	1.12 (0.04)	0.275***	-0.055	0.035	–									
5. Masculinity	3.76 (0.05)	0.230***	-0.010	0.042	0.882***	–								
6. Femininity	1.51 (0.04)	-0.240***	0.092	-0.015	-0.817***	-0.447***	–							
7. Openness	58.04 (0.66)	-0.182**	0.116†	-0.023	-0.216***	-0.115†	0.268***	–						
8. Conscientiousness	53.21 (0.74)	0.082	-0.024	-0.047	0.259***	0.230***	-0.209***	0.042	–					
9. Extroversion	53.51 (0.71)	-0.095	-0.080	-0.027	0.204***	0.229***	-0.106†	0.127*	0.427***	–				
10. Agreeableness	54.23 (0.64)	0.243***	0.046	-0.008	0.012	-0.007	-0.032	0.052	0.133*	0.142*	–			
11. Neuroticism	47.98 (0.70)	-0.183**	-0.031	-0.030	-0.347***	0.292***	0.300***	0.099	-0.471***	-0.408***	-0.210***	–		
12. TAS	49.67 (0.67)	-0.066	-0.013	-0.013	-0.335***	-0.275***	0.299***	0.055	-0.421***	-0.455***	-0.184**	0.729***	–	
13. BDI	5.97 (0.49)	0.012	-0.011	0.012	-0.187**	-0.083	0.247***	0.135†	-0.290***	-0.266***	-0.187**	0.514***	0.698***	–
14. Suicide Score	0.09 (0.02)	-0.092	-0.032	0.057	-0.209***	-0.089	0.287***	0.165**	-0.141*	-0.175**	-0.120†	0.237***	0.306***	0.493***

***p≤.001; **p≤.01; *p≤.05; †p≤.10. GRIS, Gender Role Identity Scale; TAS, Trait Anxiety Standard Score; BDI, Beck Depression Inventory. Bold values are significant.

**Table 2:** Correlation matrix for females.

Variable	*M (SE)*	Correlation coefficients												
		1.	2.	3.	4.	5.	6.	7.	8.	9.	10.	11.	12.	13.
1. Age	48.63 (0.77)	–												
2. Race	–	0.287***	–											
3. Ethnicity	–	-0.191***	-0.142**	–										
4. GRIS	-1.08 (0.03)	-0.194***	-0.010	0.023	–									
5. Masculinity	1.63 (0.03)	-0.305***	-0.074	0.060	0.796***	–								
6. Femininity	3.81 (0.03)	0.035	-0.039	0.076†	-0.825***	-0.423***	–							
7. Openness	54.35 (0.46)	-0.100*	0.044	0.024	0.177***	0.297***	-0.058	–						
8. Conscientiousness	53.25 (0.47)	0.079†	0.055	0.003	-0.136**	-0.118**	0.155***	0.073	–					
9. Extroversion	51.49 (0.44)	-0.035	0.008	0.036	-0.140**	-0.042	0.219***	0.217***	0.332***	–				
10. Agreeableness	53.95 (0.45)	0.098*	0.088†	-0.107*	-0.188***	-0.217***	0.137**	0.097*	0.264***	0.242***	–			
11. Neuroticism	45.91 (0.49)	-0.183***	-0.003	0.085†	0.129**	0.156***	-0.111*	0.040	-0.425***	-0.456***	-0.340***	–		
12. TAS	51.74 (0.56)	-0.022	0.060	-0.029	0.124**	0.108*	-0.129**	-0.022	-0.362***	-0.466***	-0.246***	0.750***	–	
13. BDI	6.38 (0.35)	-0.001	0.052	0.023	0.101*	0.141**	-0.059	-0.002	-0.231***	-0.326***	-0.215***	0.580***	0.720***	–
14. Suicide Score	0.06 (0.01)	-0.033	0.015	-0.021	0.058	0.073	-0.033	0.033	-0.067	-0.146***	-0.101*	0.272***	0.242***	0.292***

***p≤.001; **p≤.01; *p≤.05; †p≤.10. GRIS, Gender Role Identity Scale; TAS, Trait Anxiety Standard Score; BDI, Beck Depression Inventory. Bold values are significant.

Males scored higher on openness (M = 58.04, SD = 10.39), extroversion (M = 53.51, SD = 11.28), neuroticism (M = 47.98, SD = 11.11), GRIS (M = 1.12, SD = 0.57), and masculinity (M = 3.76, SD = 0.73) compared to females (M = 54.35, SD = 10.19; M = 51.49, SD = 9.80; M = 45.91, SD = 10.72; M = -1.08, SD = 0.59; M = 1.63, SD = 0.65). Females scored higher on femininity (M = 3.81, SD = 0.73 *vs*. M = 1.51, SD = 0.60) and anxiety (M = 51.74, SD = 12.26 *vs*. M = 49.67, SD = 10.61).

#### 3.1.2 Personality and mental health

##### 3.1.2.1 Males

For anxiety, regression showed a significant model (F(8, 237) = 38.165, p <.001, R²adj = 0.548) with effects of extroversion (β = -0.154, t(237) = -3.222, p = .001) and neuroticism (β = 0.609, t(237) = 12.122, p <.001). For depression, the model was significant (F(8, 197) = 10.407, p <.001, R²adj = 0.269) with neuroticism as a predictor (β = 0.291, t(197) = 6.216, p <.001). For suicidality, the model was significant (F(8, 239) = 3.331, p = .001, R²adj = 0.070) with openness (β = 0.005, t(239) = 2.552, p = .011) and neuroticism (β = 0.004, t(239) = 1.980, p = .049) as predictors (**Table 3**).

**Table 3:** Linear regressions of personality traits to predict mental health.

	Males				Females			
	TAS							
	*β*	SE	*P*	Adjusted *R*²	*β*	SE	*P*	Adjusted *R*²
				54.8%				59.1%
Age	0.015	0.028	.592		0.057	0.023	.012*	
Race	0.839	1.127	.457		0.957	0.927	.303	
Ethnicity	0.659	1.479	.657		-2.357	1.218	.054†	
Openness	0.013	0.047	.788		-0.014	0.037	.708	
Conscientiousness	-0.038	0.046	.403		-0.038	0.039	.323	
Extroversion	-0.154	0.048	.001***		-0.165	0.044	<.001***	
Agreeableness	-0.031	0.048	.513		0.023	0.040	.567	
Neuroticism	0.609	0.050	<.001***		0.807	0.042	<.001***	
	BDI							
	*β*	SE	*P*	Adjusted *R*²	*β*	SE	*P*	Adjusted *R*²
				26.9%				33.7%
Age	0.044	0.029	.127		0.026	0.021	.223	
Race	-0.937	0.997	.348		0.282	0.720	.695	
Ethnicity	1.118	1.294	.389		0.026	0.933	.978	
Openness	0.084	0.044	.057†		-0.015	0.029	.615	
Conscientiousness	-0.029	0.044	.503		0.020	0.031	.525	
Extroversion	-0.026	0.043	.538		-0.049	0.035	.166	
Agreeableness	-0.074	0.043	.086		-0.013	0.032	.685	
Neuroticism	0.291	0.047	<.001***		0.374	0.033	<.001***	
	Suicide score							
	*β*	SE	*P*	Adjusted *R*²	*β*	SE	*P*	Adjusted *R*²
				7.0%				6.7%
Age	<0.001	0.001	.737		<0.001	0.001	.872	
Race	0.008	0.050	.871		0.002	0.033	.959	
Ethnicity	0.064	0.066	.331		-0.038	0.043	.386	
Openness	0.005	0.002	.011*		0.001	0.001	.477	
Conscientiousness	<0.001	0.002	.908		0.002	0.001	.175	
Extroversion	-0.004	0.002	.094†		-0.001	0.002	.391	
Agreeableness	-0.002	0.002	.247		-0.001	0.001	.650	
Neuroticism	0.004	0.002	.049*		0.008	0.001	<.001***	

***p≤.001; **p≤.01; *p≤.05; †p≤.10. TAS, Trait Anxiety Standard Score; BDI, Beck Depression Inventory. Bold values are significant.

##### 3.1.2.2 Female

For anxiety, the model was significant (F(8, 474) = 88.145, p <.001, R²adj = 0.591) with effects of extroversion (β = -0.165, t(474) = -3.765, p <.001), neuroticism (β = 0.807, t(474) = 19.165, p <.001), and age (β = 0.057, t(474) = 2.513, p = .012). For depression, the model was significant (F(8, 403) = 27.124, p <.001, R²adj = 0.337) with neuroticism as a predictor (β = 0.374, t(403) = 11.257, p <.001). For suicidality, the model was significant (F(8, 475) = 0.416, p <.001, R²adj = 0.067) with neuroticism as a predictor (β = 0.008, t(475) = 5.035, p <.001) (**Table 3**).

### 3.2 Main analyses

#### 3.2.1 Gender roles and mental health

##### 3.2.1.1 Males

GRIS significantly predicted anxiety (F(4, 247) = 8.162, p <.001, R²adj = 0.102; β = -6.176, t(247) = -5.245, p <.001), depression (F(4, 206) = 1.971, p = .100, R²adj = 0.018; β = -2.466, t(206) = -2.750, p = .006), and suicidality (F(4, 249) = 3.249, p = .013, R²adj = 0.034; β = -0.113, t(249) = -3.013, p = .003) (**Table 4**).

**Table 4:** Linear regressions of gender roles to predict mental health.

	Males				Females			
GRIS								
	TAS							
	*β*	SE	*P*	Adjusted *R*²	*β*	SE	*P*	Adjusted *R*²
				10.2%				1.2%
Age	0.001	0.037	.972		-0.015	0.035	.665	
Race	1.547	1.557	.321		1.922	1.426	.178	
Ethnicity	0.057	2.043	.978		-1.068	1.862	.566	
GRIS	-6.176	1.177	<.001***		2.500	0.958	.009**	
	BDI							
	*β*	SE	*P*	Adjusted *R*²	*β*	SE	*P*	Adjusted *R*²
				1.8%				0.4%
Age	0.016	0.032	.620		0.003	0.026	.894	
Race	0.006	1.120	.996		0.912	0.871	.295	
Ethnicity	0.569	1.458	.697		0.694	1.118	.535	
GRIS	-2.466	0.897	.006**		1.206	0.594	.043*	
	Suicide score							
	*β*	SE	*P*	Adjusted *R*²	*β*	SE	*P*	Adjusted *R*²
				3.4%				-0.3%
Age	-0.001	0.001	.549		-0.001	0.001	.495	
Race	0.028	0.049	.569		0.015	0.034	.649	
Ethnicity	0.061	0.065	.348		-0.024	0.044	.590	
GRIS	-0.113	0.038	.003**		0.025	0.023	.260	
Masculinity								
	TAS							
	*β*	SE	*P*	Adjusted *R*²	*β*	SE	*P*	Adjusted *R*²
				6.9%				0.9%
Age	-0.022	0.037	.563		-0.009	0.036	.801	
Race	2.290	1.570	.146		2.053	1.426	.151	
Ethnicity	-0.105	2.081	.960		-1.133	1.865	.544	
Masculinity	-3.813	0.909	<.001***		2.090	0.897	.020*	
	BDI							
	*β*	SE	*P*	Adjusted *R*²	*β*	SE	*P*	Adjusted *R*²
				-1.1%				1.5%
Age	0.002	0.032	.962		0.016	0.026	.546	
Race	0.416	1.126	.712		0.936	0.865	.279	
Ethnicity	0.377	1.480	.799		0.642	1.111	.564	
Masculinity	-0.824	0.707	.245		1.636	0.550	.003**	
	Suicide score							
	*β*	SE	*P*	Adjusted *R*²	*β*	SE	*P*	Adjusted *R*²
				0.4%				-0.2%
Age	-0.002	0.001	.192		<0.001	0.001	.645	
Race	0.048	0.050	.331		0.016	0.034	.625	
Ethnicity	0.051	0.066	.443		-0.024	0.044	.579	
Masculinity	-0.031	0.029	.286		0.031	0.021	.147	
Femininity								
	TAS							
	*β*	SE	*P*	Adjusted *R*²	*β*	SE	*P*	Adjusted *R*²
				7.8%				1.3%
Age	-0.012	0.038	.751		-0.028	0.035	.420	
Race	1.542	1.586	.332		1.927	1.425	.177	
Ethnicity	-0.324	2.067	.876		-0.709	1.867	.704	
Femininity	5.074	1.125	<.001***		-2.094	0.768	.007**	
	BDI							
	*β*	SE	*P*	Adjusted *R*²	*β*	SE	*P*	Adjusted *R*²
				4.4%				-0.2%
Age	0.014	0.031	.646		-0.005	0.025	.853	
Race	-0.200	1.107	.857		0.966	0.873	.269	
Ethnicity	0.513	1.436	.721		0.789	1.127	.484	
Femininity	2.937	0.803	<.001***		-0.582	0.484	.230	
	Suicide score							
	*β*	SE	*P*	Adjusted *R*²	*β*	SE	*P*	Adjusted *R*²
				7.2%				-0.5%
Age	<0.001	0.001	.757		-0.001	0.001	.377	
Race	0.012	0.049	.812		0.016	0.034	.631	
Ethnicity	0.061	0.064	.343		-0.022	0.044	.619	
Femininity	0.153	0.035	<.001***		-0.012	0.018	.525	

***p≤.001; **p≤.01; *p≤.05; †p≤.10. GRIS, Gender role Identity Scale; TAS, Trait Anxiety Standard Score; BDI, Beck Depression Inventory. Bold values are significant.

Masculinity predicted anxiety (F(4, 247) = 5.643, p <.001, R²adj = 0.069; β = -3.813, t(247) = -4.198, p <.001) but not depression (F(4, 206) = 0.417, p = .796) or suicidality (F(4, 249) = 1.237, p = .296). Femininity predicted anxiety (F(4, 247) = 6.335, p <.001, R²adj = 0.078; β = 5.074, t(247) = 4.510, p <.001), depression (F(4, 206) = 3.429, p = .010, R²adj = 0.044; β = 2.937, t(206) = 3.659, p <.001), and suicidality (F(4, 249) = 5.872, p <.001, R²adj = 0.072; β = 0.153, t(249) = 4.405, p <.001) (**Table 4**).

##### 3.2.1.2 Females

GRIS predicted anxiety (F(4, 481) = 2.449, p = .045, R²adj = 0.012; β = 2.500, t(481) = 2.611, p = .009) and depression (F(4, 410) = 1.425, p = .225; β = 1.206, t(410) = 2.029, p = .043), but not suicidality (F(4, 482) = 0.601, p = .662) (**Table 4**).

Masculinity predicted anxiety (F(4, 481) = 2.100, p <.001, R²adj = 0.009; β = -3.813, t(481) = 2.330, p <.001) and depression (F(4, 410) = 2.610, p = .035, R²adj = 0.015; β = 1.636, t(410) = 2.973, p = .003), but not suicidality (F(4, 482) = 0.812, p = .518). Femininity predicted anxiety (F(4, 481) = 2.605, p = .035, R²adj = 0.013; β = -2.094, t(481) = -2.727, p = .007), but not depression (F(4, 410) = 0.755, p = .555) or suicidality (F(4, 482) = 0.385, p = .820) (**Table 4**).

#### 3.2.2 Moderation analyses

**Figure 2:**
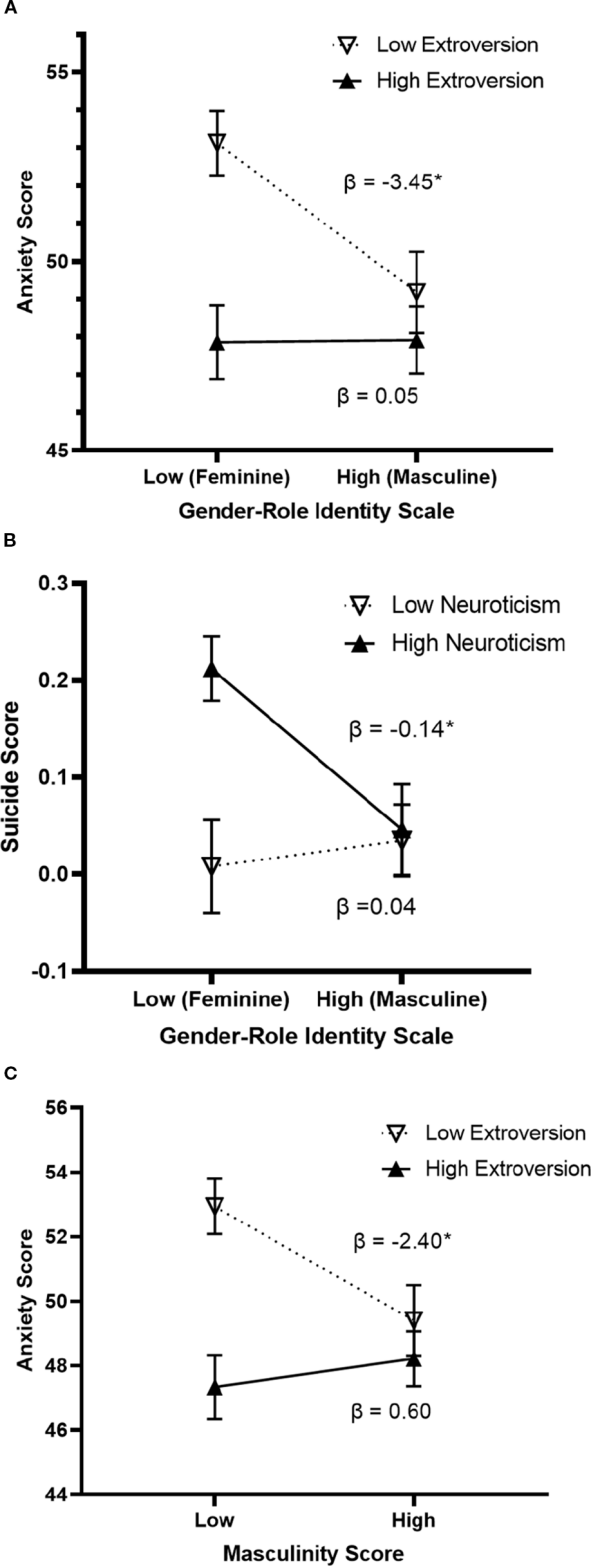
Moderation effect of personality traits on the relationship between gender roles and mental health among males (N = 254). Graph **(A)** Difference in anxiety scores between feminine and masculine males is only significant for males with low levels of extroversion. Graph **(B)** Difference in suicide scores between feminine and masculine males is only significant for males with high levels of neuroticism. Graph **(C)** Difference in anxiety scores between less masculine and more masculine males is only significant for males with low levels of extroversion *p≤.05.

Moderation (**Table 5**) showed extroversion moderated the effect of GRIS on anxiety in males (t(243) = 2.194, p = .029, ΔR² = 0.008), with GRIS predicting anxiety only at low extroversion (β = −3.450, 95% CI [-5.816, -1.084], p = .004). Extroversion also moderated masculinity’s effect on anxiety (t(243) = 2.391, p = .018, ΔR² = 0.010), significant only at low extroversion (β = -2.398, 95% CI [-4.264, -0.533], p = .012). Neuroticism moderated the GRIS–suicidality relation (t(240) = -2.554, p = .011, ΔR² = 0.024), with GRIS predicting suicidality only at high neuroticism (β = -0.136, 95% CI [-0.228, -0.043], p = .004). No significant moderation effects were found in females (**Table 6**) (**Figure 2**).

**Table 5:** Moderation analyses to predict mental health among males.

	TAS			
	*β*	SE	*P*	Adjusted *R*²
Neuroticism				
GRIS				57.1%
Age	0.027	0.027	.321	
Race	0.483	1.097	.660	
Ethnicity	0.868	1.456	.552	
Neuroticism	0.601	0.047	<.001***	
Extroversion	-0.160	0.045	<.001***	
GRIS	-1.454	0.890	.103	
GRIS x Neuroticism	-0.067	0.067	.316	
Masculinity				56.8%
Age	0.020	0.027	.454	
Race	0.637	1.095	.561	
Ethnicity	0.802	1.463	.548	
Neuroticism	0.612	0.047	<.001***	
Extroversion	-0.163	0.045	<.001***	
Masculinity	-0.683	0.661	.302	
Masculinity x Neuroticism	-0.061	0.053	.252	
Femininity				57.1%
Age	0.026	0.027	.339	
Race	0.450	1.100	.683	
Ethnicity	0.814	1.454	.576	
Neuroticism	0.602	0.047	<.001***	
Extroversion	-0.167	0.044	<.001***	
Femininity	1.514	0.826	.068†	
Femininity x Neuroticism	0.039	0.063	.537	
Extroversion				
GRIS				57.8%
Age	0.024	0.027	.383	
Race	0.586	1.086	.590	
Ethnicity	0.851	1.445	.556	
Neuroticism	0.605	0.046	<.001***	
Extroversion	-0.145	0.045	.001***	
GRIS	-1.699	0.867	.051†	
GRIS x Extroversion	0.155	0.071	.029*	
Masculinity				57.6%
Age	0.018	0.027	.499	
Race	0.770	1.080	.477	
Ethnicity	0.797	1.449	.583	
Neuroticism	0.617	0.050	<.001***	
Extroversion	-0.151	0.045	.001***	
Masculinity	-0.901	0.655	.170	
Masculinity x Extroversion	0.133	0.056	.018*	
Femininity				57.3%
Age	0.023	0.027	.396	
Race	0.500	1.099	.650	
Ethnicity	0.798	1.451	.583	
Neuroticism	0.606	0.047	<.001***	
Extroversion	-0.158	0.045	.001***	
Femininity	1.560	0.807	.054†	
Femininity x Extroversion	-0.074	0.068	.277	
	BDI			
	β	SE	P	Adjusted R²
Neuroticism				
GRIS				27.5%
Age	0.033	0.028	.240	
Race	-0.604	0.985	.540	
Ethnicity	1.129	1.294	.384	
Neuroticism	0.323	0.041	<.001***	
GRIS	-0.193	0.834	.817	
GRIS x Neuroticism	-0.062	0.063	.322	
Femininity				29.2%
Age	0.039	0.027	.151	
Race	-0.794	0.973	.415	
Ethnicity	1.184	1.277	.355	
Neuroticism	0.305	0.040	<.001***	
Femininity	1.102	0.742	.139	
Femininity x Neuroticism	0.098	0.058	.092†	
	Suicide Score			
	β	SE	P	Adjusted R²
Neuroticism				
GRIS				11.7%
Age	<0.001	0.001	.895	
Race	-0.004	0.050	.930	
Ethnicity	0.073	0.065	.265	
Neuroticism	0.005	0.002	.014*	
Openness	0.004	0.002	.050*	
GRIS	-0.049	0.040	.221	
GRIS x Neuroticism	-0.008	0.003	.011*	
Femininity				12.9%
Age	<0.001	0.001	.723	
Race	-0.010	0.049	.838	
Ethnicity	0.075	0.064	.243	
Neuroticism	0.005	0.002	.018*	
Openness	0.003	0.002	.107	
Femininity	0.104	0.037	.006**	
Femininity x Neuroticism	0.005	0.003	.088†	
Openness				
GRIS				9.5%
Age	<0.001	0.001	.892	
Race	0.002	0.050	.967	
Ethnicity	0.077	0.066	.245	
Neuroticism	0.006	0.002	.006**	
Openness	0.004	0.002	.063†	
GRIS	-0.069	0.040	.084†	
GRIS x Openness	-0.003	0.003	.470	
Femininity				12.5%
Age	<0.001	0.001	.745	
Race	-0.014	0.050	.773	
Ethnicity	0.079	0.065	.225	
Neuroticism	0.005	0.002	.008**	
Openness	0.003	0.002	.141	
Femininity	0.109	0.037	.004**	
Femininity x Openness	0.004	0.003	.180	

***p≤.001; **p≤.01; *p≤.05; †p≤.10. GRIS, Gender Role Identity Scale; TAS, Trait Anxiety Standard Score; BDI, Beck Depression Inventory. Bold values are significant.

**Table 6:** Moderation analyses to predict mental health among females.

	TAS			
	*β*	SE	*P*	Adjusted *R*²
Neuroticism				
GRIS				59.9%
Age	0.063	0.023	.007**	
Race	0.896	0.920	.330	
Ethnicity	-2.662	1.206	.028*	
Neuroticism	0.811	0.039	<.001***	
Extroversion	-0.168	0.042	<.001***	
GRIS	0.614	0.626	.328	
GRIS x Neuroticism	-0.038	0.054	.489	
Masculinity				59.9%
Age	0.064	0.024	.007**	
Race	0.932	0.919	.311	
Ethnicity	-2.586	1.202	.032*	
Neuroticism	0.809	0.039	<.001***	
Extroversion	-0.172	0.041	<.001***	
Masculinity	0.485	0.580	.404	
Masculinity x Neuroticism	-0.042	0.052	.427	
Femininity				59.8%
Age	0.060	0.023	.009**	
Race	0.936	0.921	.310	
Ethnicity	-2.538	1.207	.036*	
Neuroticism	0.810	0.039	<.001***	
Extroversion	-0.168	0.042	<.001***	
Femininity	-0.298	0.507	.557	
Femininity x Neuroticism	-0.005	0.046	.918	
Extroversion				
GRIS				59.8%
Age	0.063	0.023	.007**	
Race	0.915	0.920	.320	
Ethnicity	-2.601	1.204	.031*	
Neuroticism	0.810	0.039	<.001***	
Extroversion	-0.167	0.042	<.001***	
GRIS	0.652	0.624	.297	
GRIS x Extroversion	0.015	0.058	.804	
Masculinity				60.0%
Age	0.064	0.024	.007**	
Race	0.944	0.917	.304	
Ethnicity	-2.664	1.201	.027*	
Neuroticism	0.807	0.039	<.001***	
Extroversion	-0.176	0.041	<.001***	
Masculinity	0.440	0.579	.448	
Masculinity x Extroversion	0.087	0.058	.136	
Femininity				59.9%
Age	0.060	0.023	.008**	
Race	0.909	0.919	.323	
Ethnicity	-2.581	1.205	.033*	
Neuroticism	0.809	0.039	<.001***	
Extroversion	-0.168	0.042	<.001***	
Femininity	-0.284	0.506	.575	
Femininity x Extroversion	0.055	0.046	.224	
	BDI			
	*β*	SE	*P*	Adjusted *R*²
Neuroticism				
GRIS				34.7%
Age	0.034	0.021	.110	
Race	0.1990	0.713	.790	
Ethnicity	-0.292	0.919	.751	
Neuroticism	0.389	0.027	<.001***	
GRIS	0.578	0.491	.240	
GRIS x Neuroticism	-0.053	0.042	.209	
Masculinity				35.2%
Age	0.043	0.022	.048*	
Race	0.204	0.710	.774	
Ethnicity	-0.191	0.911	.934	
Neuroticism	0.381	0.027	<.001***	
Masculinity	1.001	0.453	.028*	
Masculinity x Neuroticism	-0.054	0.042	.194	

***p≤.001; **p≤.01; *p≤.05; †p≤.10. GRIS, Gender role Identity Scale; TAS, Trait Anxiety Standard Score; BDI, Beck Depression Inventory. Bold values are significant.

## 4 Discussion

The main objective of the present study was to investigate the associations among gender role identity and anxiety symptoms, depressive symptoms and suicidality in the general population. We found that having a gender role identity contrary to one’s birth-assigned sex was associated with a poorer overall mental health (i.e., more anxiety symptoms, depressive symptoms, and suicidality) depending on birth-assigned sex. Moreover, we found this relationship to be moderated by neuroticism and extroversion for males only.

### 4.1 Gender role identity and personality traits

Preliminary analyses highlighted that self-assessed gender roles correlated with all personality traits except agreeableness in males, and all mental health measures except suicidality in females. Anxiety symptoms, depressive symptoms, and suicidal thoughts and behaviors were also inter-correlated, consistent with the existing literature (86). Contrary to previous studies (11, 12), our analyses did not find a difference in depression scores between males and females. Surprisingly, males from our sample reported higher neuroticism levels than females, which does not align with existing literature showing the opposite pattern (87, 88). However, as expected, gender role identity scores were substantially different between males and females.

### 4.2 Gender role identity and mental health

Consistent with our hypothesis, people with a “cross-typed” or “incongruent” gender role profile (i.e., with a gender role identity opposite to their birth-assigned sex) reported poorer mental health, which is consistent with previous work (48). This is also consistent with *congruence models* of gender roles that state that gender role alignment with birth-assigned sex is less distressing (37). However, this effect turned out to be much stronger in males, where femininity was highly associated with more anxiety symptoms, depressive symptoms, and suicidality. Masculinity in females, however, was less strongly but still significantly associated with anxiety and depressive symptoms. These results are consistent with existing literature where femininity in males have been associated with poorer mental health (33, 89). Notwithstanding, these findings must expanded and replicated using approaches that assess multidimensional gender role profiles.

In contrast to males, our findings only partially align with findings regarding gender roles and mental health in females. In assessing gender roles categorically, undifferentiated females (i.e., females with low levels of masculinity and femininity) usually demonstrate higher anxiety and depression. These observations have been attributed to the low levels of masculinity which would otherwise have a protective effect (29, 37). Other research reported that higher femininity in females was associated with more anxiety (21) and depressive symptoms (39, 40). Our results suggest that it is masculinity, and not femininity, which is associated with higher levels of anxiety and depressive symptoms in females. This being said, our analyses did not allow us to explore other gender configurations (e.g., androgyny).

### 4.3 The concept of gendered societal burden

Our results and the method we used strongly suggest that several forces predict how gender role identity impacts mental health. The main force may be related to the congruence between one’s gender role identity and birth-assigned sex. Having masculine gender role identity among females and feminine gender role identity for males is linked to poorer mental health. This “gendered societal burden” may be strengthened by society’s perception which promotes sex-typed gender roles (89, 90), resulting in more stress-related issues in gender “incongruent” or non-conforming people (91). For instance, feminine gay men tend to experience anti-effeminacy prejudice (90, 92). In addition, being feminine is more likely to be associated with being perceived as gay in men than in women (93). That is, feminine men can suffer from homophobic discrimination regardless of their sexuality. In other research, homophobic discrimination impacts both anxiety and depressive symptoms above and beyond other psychological stressors. This effect is stronger among men compared to women (94). Unfortunately, sexual orientation was not ascertained in this study. Taken together, those societal issues relate to the concept of gender relations and can partially explain differences observed between males and females regarding gender roles and their relation on mental health as an elusive form of gendered societal burden related to expectations of masculine and feminine expressions. We encourage future analyses to further explore the role of sexual orientation and gender identity relative to gender role in mental health research.

### 4.4 The concept of gendered traits burden

Another force that can influence the relationship between gender roles and mental health is the nature of what characterizes personality traits as stereotypically “feminine” or “masculine”. Indeed, traits are classified as feminine and masculine based on their prevalence in each sex within a given society at a particular moment in history (25, 26). However, anxiety and depressive symptoms tend to be more prevalent in females than in males (11, 12). Overall, boys and men experience more restrictive prescriptive stereotypes than girls and women across life, and therefore variations in gender expression are more likely to be punished (95). Then, it makes sense to think that characteristics judged as “feminine” should be associated with poorer mental health. Some literature shows that this is the case (40). In this sense, this “gendered traits burden” based on gendered characteristics would not be based on birth-assigned sex per se. Instead, this could be associated with a protective effect in masculine people; however, this will need to be assessed in future studies that nuance different gender models (e.g., congruence, androgyny, gender-typed) as articulated elsewhere (37).

### 4.5 The “two-forces” hypothesis to explain literature inconsistencies

These two forces combined (i.e., gendered societal burden & gendered traits burden) may explain why feminine men in our study report poorer mental health, as well as explain why gender roles impact women’s mental health less consistently. Studies using validated questionnaires with gendered items like the BSRI and the PAQ might focus on the gendered traits burden only, whereas studies like ours may reflect a combination of both forces, especially as they relate to “cross-typed” gender role profiles. This hypothesis needs more investigation to be clearly assessed, but it underlines the importance of diversity in the methods used to assess gender roles to fully understand their relationship with mental health.

### 4.6 Personality traits and mental health

Personality traits have been strongly associated with mental health (63) independent of gender roles, but findings are inconsistent (75, 76, 96). In accordance with existing literature and our hypothesis, neuroticism appeared to be the most important personality trait predictive of mental health (63, 65). Indeed, neuroticism is the strongest factor predictive of both anxiety and suicidal thoughts and behaviors. Neuroticism is also the only significant trait to positively predict depressive symptoms for both sexes. In addition, our results suggest that extroversion is the second most important trait to predict anxiety in both males and females. Specifically, the more a person is extroverted, the less they report anxiety. A recent study also showed the best mental health profiles among androgynous individuals with high scores in both masculinity and femininity, as well as high scores in extraversion, openness to experience, emotional stability, agreeableness, and conscientiousness (97). Note, however, that results in our study are driven by feminine males and must be replicated before making any conclusions.

These results are in accordance with some studies (65). However, the present study did not find an effect of extroversion on depressive symptoms, whereas this trait has been more consistently negatively associated with depression in the literature (63, 98). Otherwise, openness was also significatively associated with suicidal thoughts and behaviors, but only in males. This result highlights that different factors affect suicidality among males and females, and suggest that personality traits, in relation to gender roles and birth-assigned sex, may in part account for the “gender paradox” (i.e. higher women suicide attempts versus higher men suicide completion (5)).

### 4.7 Neuroticism moderates the relationship between gender role identity and suicidality

As personality traits are linked to the way people feel, act, and appear to others (62), they may impact the relationship between gender role identity and mental health. Moderation analyses revealed that some personality traits moderate this relationship but only among males, where the effect of gender role identity on mental health is much stronger than in females. Specifically, neuroticism, which is strongly associated with a poorer mental health (65), moderates the effect of the gender role identity on suicidality. Interestingly, it appears that only highly neurotic males display the deleterious effect of cross-typed profile on suicidality. Indeed, males with low levels of neuroticism did not report suicidal thoughts or behaviors, neither when they were highly feminine or highly masculine. Conversely, masculine males reported low suicidality regardless of whether they were neurotic or not. Only feminine neurotic males reported significantly higher suicidal thoughts and behaviors.

### 4.8 Implications for understanding the gender paradox in mental health

Our findings highlight the protective aspect of masculinity against suicidal ideation and self-injury in males that have been consistently reported in the literature (53, 58). Furthermore, they bring a new perspective to the “gender paradox” in mental health. In summary, our results suggest that it is important to consider the core relationships among gender roles, personality traits, and birth-assigned sex collectively when endeavoring to explain gender differences in suicidality. This underlines the fact that it is not only women who display more suicidal thoughts and behaviors, but also men with specific characteristics (i.e., feminine and neurotic). One caveat is that our sample was composed of living people, which limits us from making conclusions about gender roles in relation to suicide completion that is an essential endpoint of the “gender paradox” in suicide. From this perspective, specific gender roles and behaviors may indeed influence some suicide-related characteristics, like the use of lethal means that have been proposed to explain why men complete more suicide than women (99, 100). Further research is required to fully understand the impact of specific gender roles in relation to suicide completion to explain higher prevalence in men.

### 4.9 Extroversion moderates the relationship between gender role identity and anxiety

On the other hand, our analyses revealed that extroversion moderates the relation between gender role identity and anxiety. A similar moderation effect was detected for masculinity alone. It appears that extroverted people (i.e., with high levels of extroversion) have low anxiety scores, regardless of whether they are feminine or masculine. Gender role identity differences in anxiety were only observed in males with low levels of extroversion. Taken together, only low-extroverted feminine males reported significantly higher levels of anxiety. Extroversion is a trait that depicts our relationship to others the most by “initiating social contacts” (101). In this sense, by expressing their femininity, more extroverted feminine males may be able to experience anxiety levels more akin to masculine males, thwarting the deleterious effect of their cross-typed profile. Conversely, feminine characteristics of males that are less extroverted may exacerbate their anxiety by concealing a part of them that they perceive society reprimands and censors. This hypothesis needs to be more thoroughly investigated in future studies in cross-cultural contexts.

Similar to our first moderation analysis described above, these results put forward the protective effect of masculinity against anxiety which has been consistently observed in the literature (21, 29, 36, 102). However, our results underline the importance of considering personality traits to better understand the circumstances in which this protective effect manifests itself in diverse contexts. Interestingly, personality traits that moderate the relationship between gender role profiles and mental health are not the same depending on the psychiatric symptom or construct being studied. Considering that anxiety is an important risk factor for suicidal behaviors (10, 103), our study highlights that restricting research to gender roles and mental health without taking into account the influence of different personality traits might miss important connections with mental health.

### 4.10 Limitations

The present study has limitations worth discussing. In contrast to previous studies that assessed gender roles using inventories of gendered personality traits (e.g., the Bex Sex Role Inventory, Personal Attributes Questionnaire), participants directly assessed their gender roles broadly. Other gender role instruments were also originally constructed to assess adaptive androgyny defined as high masculinity and high femininity. As gender roles can depend on society and how people are socialized, the concepts of what is deemed feminine/masculine may differ from one culture to another. Even if recruiting people from the same geographic location (i.e., Rockland town) might reduce confounding, further studies should be conducted in areas where conceptualizations of masculinity and femininity are different to assess if these results generalize to other cultures and environments.

Furthermore, the present study only considered gender role identity as a continuum. As such, results are limited to cross-typed and sex-typed profiles (i.e., feminine and masculine people), without looking at people presenting high or low levels of both masculinity and femininity (i.e., androgynous and undifferentiated people). To address this limitation, we conducted analyses with both continuous and categorical approaches (data not shown). Unfortunately, categorical approaches did not provide more information than the continuous approach we reported, so we did not include these in our report. This absence of significant differences may be due to the measurement methods. Indeed, gender roles can be conceptualized along a single continuum when participants self-report (e.g., being more masculine will decrease their femininity), resulting in a limitation of the number of androgenous and undifferentiated profiles. This is in contrast to Bem’s theory that masculinity and femininity are two independent continuums; however, this assumption has nothing to do with how people self-report with their own personal assumptions of their gender role identity.

Another limitation is that the present study considered suicidality as a continuum from no suicidal thoughts or behaviors to the presence of both. This decision was made with analytic consideration for the low rate of suicidal behaviors in the studied sample from general population. Suicide scores were then analyzed as a continuous variable, preventing the dissociation between suicidal thoughts only and suicidal behaviors. However, literature has shown that risk factors for suicidal ideations differ from those for suicide attempt (104). In addition, the current study cannot provide information for the relationship between gender roles, personality traits, and suicide completion. Finally, even if the present study emphasizes the effect of gender role identity and personality traits on mental health, it does not assess which factors cause or influence the other. Another issue is potential confounding related to neuroticism that is associated with anxiety and depression (105) while also interacting with gendered behaviors (106). Further longitudinal studies and mediation analysis need to be conducted to address causal pathways, especially with designs assessing lifespan development.

## 5 Conclusion

Our study offers new insight into the complex interplay of gender roles, personality, and mental health. Self-assessed masculinity and femininity predict anxious, depressive, and suicidal symptoms, but in sex-specific ways. Notably, adopting a gender role contrary to birth-assigned sex is linked to poorer mental health, with effects especially pronounced in males. This suggests that both cross-typed profiles and feminine characteristics heighten vulnerability to distress in men. Personality further shapes these dynamics: only neurotic feminine males reported greater suicidal symptoms, and only less extroverted feminine males reported higher anxiety. These findings underscore the need to account for personality traits alongside sex and gender roles in mental health research, and to pursue further studies clarifying the mechanisms underlying these associations.

## Data Availability

Publicly available datasets were analyzed in this study. This data can be found here: Nathan Kline Rockland Sample (https://rocklandsample.org).
