# Supplementary material for: Gender role identity, personality factors, and psychiatric symptoms among American adults: the Nathan Kline Institute Rockland Sample
Source: Front Psychiatry. 2025 Sep 26;16:1594762. doi: 10.3389/fpsyt.2025.1594762 (PMC12510924; doi:10.3389/fpsyt.2025.1594762)
Supplement: Supplementary file 1 [file DataSheet1.pdf]

Supplemental Results

MANOVAs revealed a significant effect of gender role identity scores on most personality traits for both males and females (See Supplemental Table 1.).

Males

MANOVAs revealed a significant effect of GRIS on personality traits ($F_{(5, 242)} = 10.421$, $p < .001$, $\eta^2_p = 0.177$). Particularly, GRIS had a significant effect on the levels of openness ($F_{(1, 246)} = 12.032$, $p = .001$, $\eta^2_p = 0.047$), conscientiousness ($F_{(1, 246)} = 16.743$, $p < .001$, $\eta^2_p = 0.064$), extroversion ($F_{(1, 246)} = 10.104$, $p = .002$, $\eta^2_p = .039$), and neuroticism ($F_{(1, 246)} = 33.688$, $p < .001$, $\eta^2_p = 0.120$), but not on agreeableness ($F_{(1, 246)} = 10.104$, $p = .980$, $\eta^2_p < 0.001$). MANOVAs also revealed a significant effect of masculinity scores on personality traits ($F_{(5, 242)} = 6.802$, $p < .001$, $\eta^2_p = 0.123$). Particularly, masculinity had a significant effect on the levels of conscientiousness ($F_{(1, 246)} = 13.282$, $p < .001$, $\eta^2_p = 0.051$), extroversion ($F_{(1, 246)} = 12.896$, $p < .001$, $\eta^2_p = 0.050$), and neuroticism ($F_{(1, 246)} = 22.671$, $p < .001$, $\eta^2_p = 0.084$), but not on openness ($F_{(1, 246)} = 3.282$, $p = .071$, $\eta^2_p = 0.013$), nor on agreeableness ($F_{(1, 246)} = 11.952$, $p = .734$, $\eta^2_p < 0.001$). Finally, MANOVAs revealed a significant effect of femininity on personality traits ($F_{(5, 242)} = 9.023$, $p < .001$, $\eta^2_p = 0.157$). More precisely, femininity had a significant effect on the levels of openness ($F_{(1, 246)} = 19.081$, $p < .001$, $\eta^2_p = 0.072$), conscientiousness ($F_{(1, 246)} = 10.370$, $p = .001$, $\eta^2_p = 0.040$), and neuroticism ($F_{(1, 246)} = 24.655$, $p < .001$, $\eta^2_p = 0.091$), but not on extroversion ($F_{(1, 246)} = 2.652$, $p = .105$, $\eta^2_p = 0.011$), nor on agreeableness ($F_{(1, 246)} = 24.655$, $p < .001$, $\eta^2_p = 0.091$).

Supplemental Tables

Females

MANOVAs revealed significant effect of GRIS on personality traits ($F_{(5, 478)} = 9.944$, $p < .001$, $\eta^2_p = 0.094$). Particularly, GRIS had a significant effect on the levels of every personality trait : openness ($F_{(1, 482)} = 14.864$, $p < .001$, $\eta^2_p = 0.030$), conscientiousness ($F_{(1, 482)} = 8.465$, $p = .004$, $\eta^2_p = .017$), extroversion ($F_{(1, 482)} = 8.440$, $p = .004$, $\eta^2_p = 0.017$), agreeableness ($F_{(1, 482)} = 17.678$, $p < .001$, $\eta^2_p = 0.035$), and neuroticism ($F_{(1, 482)} = 7.169$, $p = .008$, $\eta^2_p = 0.015$). MANOVAs also revealed a significant effect of masculinity on personality traits ($F_{(5, 478)} = 17.750$, $p < .001$, $\eta^2_p = 0.157$). Particularly, masculinity had a significant effect on the levels of openness ($F_{(1, 482)} = 46.236$, $p < .001$, $\eta^2_p = 0.088$), conscientiousness ($F_{(1, 482)} = 6.653$, $p = .010$, $\eta^2_p = 0.014$), agreeableness ($F_{(1, 482)} = 23.905$, $p < .001$, $\eta^2_p = 0.047$), and neuroticism ($F_{(1, 482)} = 11.673$, $p = .001$, $\eta^2_p = 0.024$), but not on extroversion ($F_{(1, 482)} = 0.696$, $p = .405$, $\eta^2_p = 0.001$). Finally, MANOVAs revealed a significant effect of personality traits ($F_{(5, 478)} = 7.286$, $p < .001$, $\eta^2_p = 0.071$). More precisely, femininity had a significant effect on the levels of conscientiousness ($F_{(1, 482)} = 10.900$, $p = .001$, $\eta^2_p = .022$), extroversion ($F_{(1, 482)} = 22.900$, $p < .001$, $\eta^2_p = 0.044$), agreeableness ($F_{(1, 482)} = 9.227$, $p = .003$, $\eta^2_p = 0.019$), and neuroticism ($F_{(1, 482)} = 4.846$, $p = .028$, $\eta^2_p = 0.010$), but not on openness ($F_{(1, 482)} = 1.277$, $p = .259$, $\eta^2_p = 0.003$).

Supplemental Tables

Supplemental Table 1. MANOVAs of gender roles to predict personality traits

		Males				Females			
Independent variable	Dependent variable	*β*	SE	*P*	Adjusted R^2	*β*	SE	*P*	Adjusted R^2
**GRIS**	Openness	-3.934	1.601	**.001*** **	4.3%	2.981	0.773	**<.001*** **	2.8%
	Conscientiousness	5.232	1.279	**<.001*** **	6.0%	-2.317	0.796	**.004** **	1.5%
	Extroversion	3.924	1.234	**.002* **	3.6%	-2.157	0.742	**.004** **	1.5%
	Agreeableness	0.029	1.133	.980	-0.4%	-3.126	0.743	**<.001*** **	3.3%
	Neuroticism	-6.767	1.166	**<.001*** **	11.7%	2.187	0.817	**.008** **	1.3%
**Masculinity**	Openness	-1.620	0.894	.071†	0.9%	4.628	0.681	**<.001*** **	8.6%
	Conscientiousness	3.636	0.997	**<.001*** **	4.7%	-1.868	0.724	**.010** **	1.2%
	Extroversion	3.417	0.951	**<.001*** **	4.6%	-0.567	0.679	.405	-0.1%
	Agreeableness	-0.299	0.878	.734	-0.4%	-3.279	0.671	**<.001*** **	4.5%
	Neuroticism	-4.390	0.922	**<.001*** **	8.1%	2.522	0.738	**.001*** **	2.2%
**Femininity**	Openness	4.621	1.058	**<.001*** **	6.8%	-0.724	0.641	.259	0.1%
	Conscientiousness	-3.940	1.224	**.001*** **	3.7%	2.144	0.649	**.001*** **	2.0%
	Extroversion	-1.929	1.184	.105	0.7%	2.808	0.599	**<.001*** **	4.2%
	Agreeableness	-0.496	1.070	.644	-0.3%	1.862	0.613	**.003** **	1.7%
	Neuroticism	5.563	1.120	**<.001*** **	8.7%	-1.473	0.669	**.028* **	0.8%

Note: ***p≤.001; **p≤.01; *p≤.05; †p≤.10. GRIS, Gender Role Identity Scale.
